# Effect arylamine N-acetyltransferase 1 on morphology, adhesion, migration, and invasion of MDA-MB-231 cells: role of matrix metalloproteinases and integrin αV

**DOI:** 10.1080/19336918.2019.1710015

**Published:** 2020-01-07

**Authors:** Pengcheng Li, Neville J. Butcher, Rodney F. Minchin

**Affiliations:** School of Biomedical Sciences, University of Queensland, St Lucia, Australia

**Keywords:** NAT1, arylamine, migration, adherence, invasion, integrin. MMP9

## Abstract

Reducted arylamine N-acetyltransferase (NAT1) in breast cancers is associated with poor patient survival. NAT1 has also been associated with changes in cancer cell survival and invasion both *in* *vitro* and *in* *vivo*. Here, we report the effects of NAT1 in cancer cell invasion by addressing its role in adherence, migration, and invasion *in vitro*. The NAT1 gene was deleted in MDA-MB-231, HT-29 and HeLa cells using CRISPR/Cas9 gene editing. Loss of NAT1 increased adherence to collagen in all three cell-lines but migration was unaffected. NAT1 deletion decreased invasion and induced changes to cell morphology. These effects were independent of matrix metalloproteinases but were related to integrin ITGαV expression. The data suggest NAT1 is important in adhesion and invasion through integrin expression.

## Introduction

Breast cancer is a heterogeneous disease derive from about five distinct cell populations that vary in proliferative and invasive capacity, recurrence, and treatment response [1]. The genetic differences between breast cancer sub-populations have been used to guide new treatment protocols that have markedly improved patient survival [2]. There are now at least 33 gene signatures for breast cancer classification that identify patients at high and low risk of recurrence or patients likely to respond to neoadjuvant chemotherapy [3]. Amongst some of these signatures is the human arylamine N-acetyltransferase 1 (NAT1) gene, which encodes a drug metabolizing enzyme widely expression in the body. In breast cancer, NAT1 expression is inversely related to overall survival and predicts response to cytotoxic chemotherapy but not hormonal therapy [4]. These observations have led several investigators to suggest a role for NAT1 in cell biology independent of its drug metabolizing functions. However, the molecular and cellular effects of NAT1 expression in breast cancer remain to be completely understood.

Inhibition of NAT1 activity in cancer cells either by siRNA-mediated silencing, small molecular inhibitors or gene deletion with CRISPR/Cas 9 editing results in changes in cell survival and invasion [5–9]. Deletion of NAT1 has been associated with epithelial-mesenchymal plasticity [7], a hallmark of invasive potential [10]. Moreover, NAT1 is required for invasion in a mouse model of breast cancer pulmonary metastases [9] suggesting it may be a druggable target in some cancers. However, survival data in patients suggest a different role for NAT1. In breast cancer, survival is significantly better in those patients with high tumor NAT1 expression compared to those with low tumor NAT1 expression [4].

MDA-MB-231 cells are triple-negative breast cancer cells with a spindle-like morphology. These cells lack E-cadherin expression and readily metastasis in animal models suggesting a mesenchymal-like phenotype [11,12]. Recently, it was demonstrated that deletion of NAT1 in MDA-MB-231 cells up-regulated MMP expression, in particular MMP9 [13]. This change was also seen in the highly invasive estrogen receptor-positive T-47D breast carcinoma cells. Moreover, gene expression in patient tumor samples showed a significant inverse relationship between NAT1 expression and MMP9 expression. The association between MMP expression and cancer cell invasion is well documented and this has been demonstrated for MDA-MB-231 cells [14,15]. Consequently, the up-regulation of MMP9 following NAT1 deletion is not in agreement with the reported decrease in invasion following NAT1 inhibition in these cells [9]. However, MDA-MB-231 cells also demonstrate integrin-dependent amoeboid motility in 3-dimensional matrigel invasion assays [16] and the possible effects of NAT1 on integrins have not been reported. In the present study, the role of MMPs and integrins in MDA-MB-231 cell adherence, migration and invasion following NAT1 deletion was studied using real-time cell motility analysis. This approach provides temporal information about cell motility through different matrices and can be used to monitor changes when MMP and/or integrin function changes. The results may explain the apparent dichotomy between NAT1 deletion, MMP expression and cell invasion.

## Results

**Figure 1.**
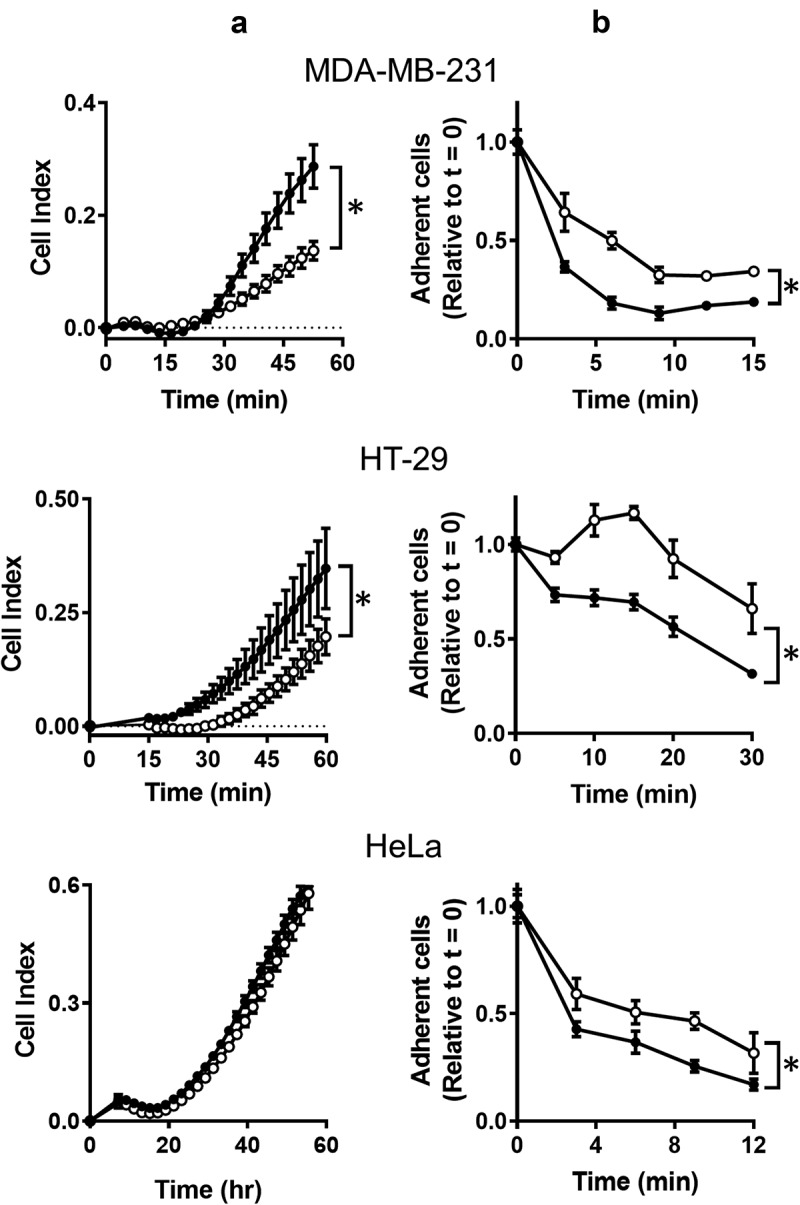
The effect of NAT1 deletion on cell adhesion. (a) Adherence of MDA-MB-231, HT-29 and HeLa cells was quantified in a xCELLigence RTCA DP analyser by increased impedance as cell adhered to each well (cell index). Parental cells (●); KO cells (〇). (b) Removal of cells from plastic culture plate with 0.05% trypsin. Data are presented as mean ± sem, n = 4. Asterisk indicates p < 0.05 by two-way ANOVA.

One characteristic observed while culturing NAT1 KO cells is their ability to adhere to plastic culture dishes and their relative resistance to removal with trypsin suggesting adherence is affected by NAT1 deletion. Cell adherence was therefore measured using two different assays. In the first assay, the rate of adhesion was quantified. MDA-MB-231, HT-29 and HeLa parental and KO cells were added to the xCELLigence RTCA DP analyzer in wells coated with collagen and impedance was measured over 60 min (Figure 1a). An increase in impedance (cell index) is indicative of increased cell adhesion. For both MDA-MB-231 and HT-29 cells, the parental lines adhered to collagen more rapidly than the NAT1 knockout line. By contrast, no difference was seen in the HeLa cells. The second assay measured the rate of cell detachment from a substrate using trypsin. Cells were cultured on serum-treated polystyrene culture plates over 24 hr, which induces focal adhesion formations between the cells and substrate-bound vitronectin derived from the serum [17]. For all three cell-lines, trypsin detached the parental cells more rapidly than the NAT1 knockout cells (Figure 1b). Taken together, these assays suggest a difference in the adhesion and detachment of cells following NAT1 deletion.

**Figure 2.**
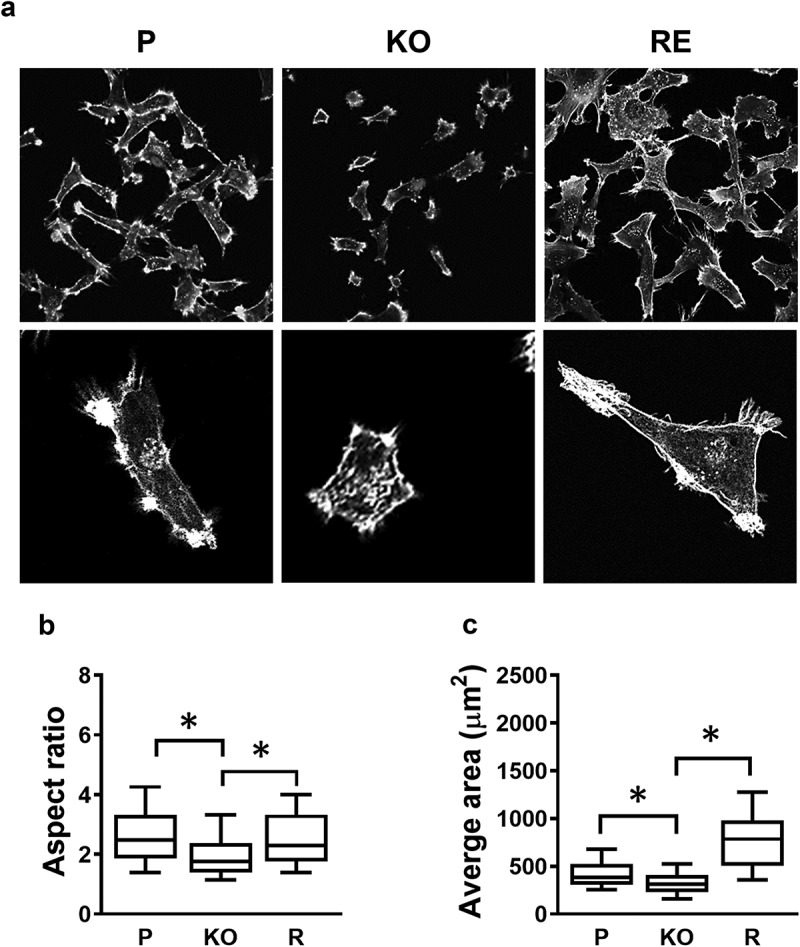
Changes in cell morphology following NAT1 deletion. (a) Confocal images of MDA-MB-231 parent (p), NAT1 knock out (KO) and NAT1 rescue (r) cells cultured on collagen for 20 hr, fixed and stained with phalloidin. Quantification of aspect ratio (b) and cell size (c) for each of the cell lines using ImageJ. Results are shown as mean with 10–90% range, n > 60. Asterisks p < 0.05 by one way ANOVA with Tukey’s multiple comparisons test.

Because the rate of adherence to collagen was very different between the parental and NAT1 knockout cells, the morphology of each was assessed following culture of MDA-MB-231 cells for 24 hr on collagen-coated coverslips. (Figure 2a). There were clear morphological differences, highlighted by both size and shape, when NAT1 was knocked out, which was recovered when NAT1 was rescued. The aspect ratio, a measure of cell roundedness, significantly decreased following NAT1 deletion (Figure 2b). Similarly, the average area was less in the knockout cells compared to the parental cells (Figure 2c).

**Figure 3.**
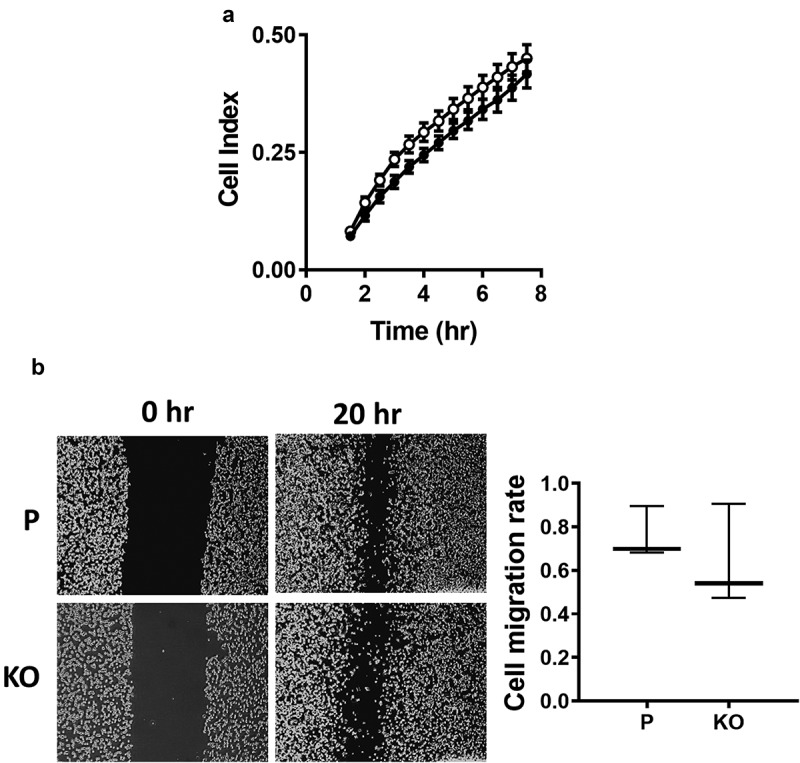
Effect of NAT1 deletion on migration in MDA-MB-231 cells. (a) Real-time measurement of MDA-MB-231 parent parental cells (●) and NAT1 KO cells (〇) migration. (b) Wound healing assay was performed in both parental (p) and NAT1 knockout (KO) cells over 20 hr. Migration was quantified and is expressed as mean with 10–90% range, n = 3.

Migration of the MDA-MB-231 cells was measured using real-time impedance analysis as well as a wound healing assay. In the first experiment, the rate of cell migration was determined following cell adhesion. There was no significant difference between the parental and NAT1 deleted cells (Figure 3a). Similarly in the wound healing assay, there was no difference between the cell-lines. Collectively, these experiments indicated that NAT1 deletion does not affect the ability for MDA-MB-231 cells to migrate.

**Figure 4.**
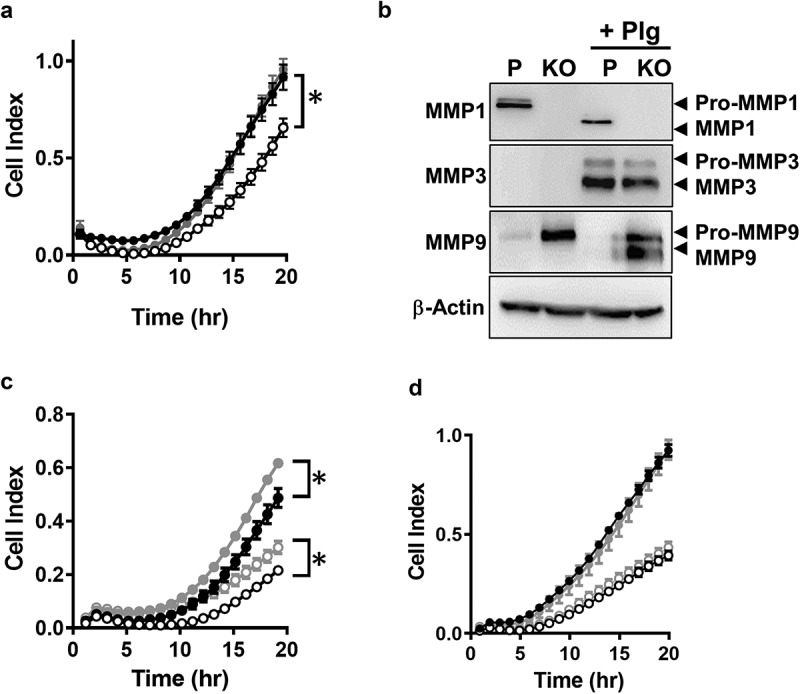
Effect of MMP expression on MDA-MB-231 invasion through matrigel. (a) Real time monitoring of invasion for parental (●), NAT1 KO cells (〇) and NAT1 rescue (●) cells Asterisk indicates p < 0.05 by two-way ANOVA. (b) Expression of MMP1, 3 and 9 in NAT1 parental (p) and knockout (KO) cells in the absence or presence (+Plg) of plasminogen. Western blots are representative of at least 2 independent experiments. (c) Effect of plasminogen on the invasion of parental ● – plasminogen; ● + plasminogen) and NAT1 KO cells (〇 – plasminogen; 〇+ plasminogen). Asterisk indicates p < 0.05 by two-way ANOVA. (d) Effect of MMP pan inhibitor GM6001on the invasion of parental (● – GM6001; ● + GM6001) and NAT1 KO cells (〇 – GM6001; 〇+ GM6001). Results are shown as mean ± sem, n = 4.

NAT1 knockdown in MDA-MD-231 cells with lentivirus to 25% of parental cells resulted in a significant decrease in invasion through matrigel [8]. By contrast, when activity was decreased by 39%, no change in invasion was seen [6]. The role of NAT1 in cell invasion *in vitro* was revisited using CRISPR/Cas 9 gene editing to ensure complete reduction in NAT1 expression. Similar to previous reports, NAT1 deletion significantly decreased cell invasion (Figure 4a). Moreover, when NAT1 was rescued, invasion returned to that seen in the parental cells. These experiments were performed using serum as a chemoattractant in the lower chamber of the real-time monitoring apparatus. In the upper chamber, cells were seeded in the absence of serum. Secreted MMPs require serum-derived proteases for activation and the absence of serum could explain why NAT1 deleted cells secrete more MMP9 than parental cells but show attenuated invasion. To test this, secreted MMP1, 3 and 9 were measured by Western blotting (Figure 4b). Full blots are shown in Supplementary figure. Pro-MMP1 was down-regulated in the NAT1 knockout cells whereas Pro-MMP9 was up-regulated, consistent with previously published results [13]. There was no evidence for MMP3 secretion in either cell-line. In addition, there was no evidence that the MMPs were activated in the absence of serum. To cleave the secreted MMPs, plasminogen was added to the cell cultures for 24 hr. Plasminogen is activated to plasmin by urokinase-type plasminogen activator, which is highly expressed in MDA-MB-231 cells [18]. Plasmin is able to activate multiple MMPs [19,20]. Addition of plasminogen resulted in complete activation of MMP1 in the parental cells and partial activation of MMP9 in the knockout cells. Plasminogen also induced MMP3 secretion equally in both cell-lines (Figure 4b).

To determine whether MMP activation affects invasion, cells cultured in the presence and absence of plasminogen were monitored for their ability to migrate through matrigel. Plasminogen slightly increased the invasive capacity of both the parental and NAT1 deleted cells (Figure 4c). However, it did not overcome the attenuated invasion seen in the knockout cell-line. Finally, because there may be other MMPs involved in MDA-MB-231 invasion, a pan MMP inhibitor (GM6001) was used in the invasion assay (Figure 4d). There was no difference in invasion of either parental or NAT1 knockout cells following treatment. These results suggest that the MMPs do not contribute significantly to the invasion of MDA-MB-231 cells through the matrigel substrate used in this and other studies.

**Figure 5.**
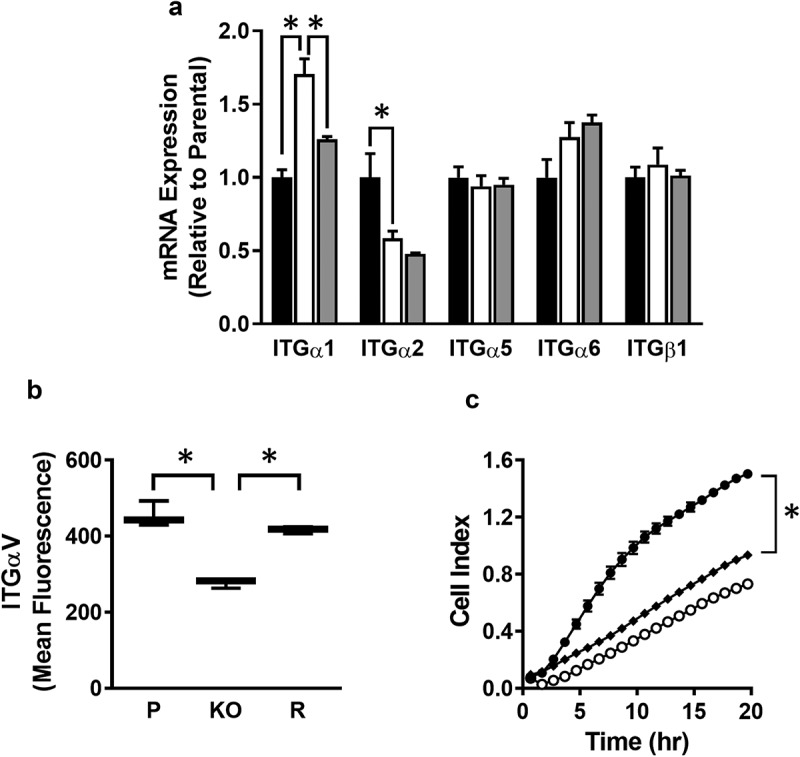
Role of integrins in MDA-MB-231 invasion (a) Integrin expression in parental (black bar), NAT1 knockout (open bar) and rescue (grey bar) cells. Results are shown as mean ± sem, n = 3. Asterisks p < 0.05 by one way ANOVA with Tukey’s multiple comparisons test. (b) Quantification of ITGαV surface expression in parental (P), NAT1 knockout (KO) and NAT1 rescue (R) cells lines. Results are shown as mean with 10–90% range, n = 4. Asterisks p < 0.05 by one way ANOVA with Tukey’s multiple comparisons test. (c) Effect of ITGαV antibody treatment on the invasion of parental cells compared to NAT1 KO cells. Parental (●), NAT1 KO cells (〇) and parental plus ITGαV antibody (♦). Results are shown as mean ± sem, n = 4. Asterisk indicates p < 0.05 by two-way ANOVA.

MMP-independent mechanisms have been proposed for breast cancer cell invasion, including integrin-dependent amoeboid motility. Integrins are also involved in cell adhesion. Expression of the major integrins in MDA-MB-231 cells was quantified by qPCR and is shown in Figure 5a. There was a significant increase in ITGα1 in the NAT1 deleted cells, which was rescued when NAT1 was re-introduced. By contrast, there was a decrease in ITGα2 expression, but this was not rescued, suggesting the change was NAT1-independent. All of the other integrins showed similar expression in all three cell-lines. These results do not explain the reduction in invasion following NAT1 deletion, although the increase in ITGα1 is consistent with greater adhesion in the knockout cells.

In addition to those integrins shown in Figure 5a, others have been associated with breast cancer invasion. Of particular interest is ITGαV, which is a marker of the mesenchymal phenotype in breast cancer cells and is highly expressed in MDA-MB-231 cells [21]. Cell surface ITGαV, quantified by flow cytometry, was significantly decreased in the NAT1 knockout cells, but was the same as the parental cells and the rescue line (Figure 5b). To determine whether this decrease might account for the change in invasiveness, parental cells were treated with an anti-ITGαV antibody to block its function. Figure 5c shows that treatment decrease invasion to approximately the same as that seen in the NAT1 knockout cells. These results suggest changes in cell surface ITGαV expression may contribute to a decrease in cell invasion.

## Discussion

The most striking finding from the current study is the apparent lack of involvement of the MMPs in NAT1-dependent invasion of MDA-MB-231 cells. This was demonstrated by the absence of activated MMPs in the medium during the invasion assay, the minor effect that MMP activation by plasminogen produced in both parental and NAT1 deleted cells, and the lack of any effect of the pan-MMP inhibitor GM6001 on invasion. The results also show that the previously reported increase in MMP9 expression following NAT1 knockout [13] is unrelated to invasion. Nevertheless, there is strong evidence of an association between MMP expression and breast cancer invasion and metastasis in patients, including MMP9 [22]. However, clinical trials with numerous MMP inhibitors have shown little or no survival benefit and severe side-effects in cancer patients [22,23]. The failure of MMP inhibitors may, in part, be due to a lack of understanding of when or how MMPs promote migration and invasion in different cancers. Alternatively, it may reflect other pathways that are equally or more important in tumor cell metastasis. In 3-dimensional invasion assays, MDA-MB-231 cell invasion was classified as protease-independent but adhesion-dependent, which is distinct from the typical adhesion independent amoeboid cell motility seen in many cancer cells [16]. This is consistent with other studies into the mechanism of MMP-independent invasion for MDA-MB-231 cells [16,24,25]. If the results reported here for the highly metastatic MDA-MB-231 cells are applicable to other invasive breast cancers, then MMP inhibition will not be an appropriate strategy in these patients.

Inhibition of NAT1 expression in colon carcinoma HT-29 and prostate carcinoma cells 22Rv1 cells up-regulated e-cadherin, a marker of a more epithelial phenotype [7]. Similarly, NAT1 deletion in MDA-MB-231 cells was associated with an increase in Snail expression and a loss of N-cadherin and β-catenin expression, which also suggests a more epithelial phenotype in these cells [9]. Mesenchymal-epithelial plasticity also involves changes in integrin expression [26]. The integrins are adhesion molecules that transmit information between the extracellular matrix, the cytoskeleton of the cells and various signalling pathways [27]. In the current study, there were no changes in the major integrins that could easily explain the change in cell invasion. ITGα2 was significantly less in the NAT1 knockout cells, but this was not rescued when NAT1 was re-introduced. Cell surface expression of ITGαV was assessed because it heterodimerizes with many integrin β sub-units including β1, 3, 5, 6, and 8 [28]. ITGαV was down-regulated in the NAT1 deleted cells, and its surface expression was rescued. Thus, loss of ITGαV may explain the decrease in invasion as this integrin has been associated with invasion in colorectal cancer [29] and breast cancer [30]. When ITGαV was inhibited in the MDA-MB-231 cells, their invasiveness was similar to that seen in the NAT1 deleted cells. This is in agreement with previous findings demonstrating that ITGαv regulates MDA-MB-231 breast cancer invasion *in vitro*, as well as in zebrafish and mouse xenograft models [31,32]. Moreover, previous studies have shown that ITGαv modulates invasion through regulation of mesenchymal genes such as N-cadherin and Snail [31,33].

Cancer cell invasion and metastasis involves filamentous actin-dependent cytoskeletal remodeling with the formation and dissolution of focal adhesion sites. NAT1 knockdown cells demonstrate impaired filopodia formation, which suggested that cytoskeletal remodeling may occurs in the absence of NAT1 [9]. A close investigation of the size and shape of the NAT1 deleted cells confirmed distinct morphological changes that were rescued when NAT1 was re-introduced to the cells. Along with a small, more rounded morphology, the NAT1 deleted cells showed enhanced adherence to extracellular matrix proteins such as collagen. This was also seen following NAT1 deletion in HT-29 and HeLa cells. The increase in adhesion may be explained by the increase in ITGα1 seen in the NAT1 knockout cells. This integrin promotes adhesion to collagen. However, further work is required to demonstrate which integrin, if any, can explain the increased adhesion of NAT1 deleted cells.

In conclusion, we have discovered that NAT1 is required for optimal invasion of MDA-MD-231 cells the integrin signalling pathway. The ITGαV pathway is under investigation for the development of novel therapeutics for cancer with several drugs already entering clinical trials [34–36]. NAT1, if linked more broadly with ITGαV expression, may be a useful marker for patients most likely to respond to these treatments.

## Methods

### Cell culture

Human breast cancer cell line MDA-MB-23, Colon carcinoma line HT-29 and cervical carcinoma line HeLa were obtained from American Type Culture Collection (USA). Cells were maintained in RPMI-1640 medium (Thermo Fisher Scientific) supplemented with 10% FCS (Hyclone), 2mM L-glutamine and 100 U/ml penicillin-streptomycin (Thermo Fisher Scientific). Cells were cultured in the humidified incubator at 37°C with 5% CO_2_ and passaged when they reached 80–90% confluence.

### Generation of NAT1 CRISPR/Cas9 knockout cell lines

The NAT1 gene was disrupted using CRISPR/Cas9 system as previously described [37]. After selection with puromycin (5 µM), single colonies were selected, expanded, and NAT1 activities measured by a high-performance liquid chromatography assay to screen for the NAT1 knockout cell lines [38]. NAT1 knockout (KO) cells were further verified by Western blot using a NAT1 specific antibody (Abcam) and by PCR of genomic DNA showing NAT1 gene disruption [37]. The NAT1 KO cells were also stably transfected with a NAT1 vector to express NAT1 as a NAT1 rescue (R) cell line [13].

### Quantitative real-time PCR

RNA was extracted from each cell-line using RNeasy Mini Kit (Invitrogen) according to the manufacturer’s instructions. RNA concentration and purity were measured using a NanoDrop Spectrophotometer ND-1000 (Thermo Fisher Scientific). cDNA synthesis was performed using 1.5 µg RNA and SuperScript III Reverse Transcriptase kit (Invitrogen). Each cDNA reaction was diluted 1:5 with nuclease-free water. Quantitative real-time PCR was performed using a QuantStudio6 96-Well RT PCR system with SensiFast SYBR Green (Bioline, Australia), with the following conditions: enzyme activation at 95ºC for 10 min, followed by 40 cycles of denaturation at 95ºC for 15 sec, annealing and extension at 60ºC for 30 sec. Target genes were amplified using specific primers (Table 1). The specificity of amplified genes was confirmed by running the PCR products on 2% agarose gels.

**Table 1.** Primers used for qPCR experiments.

Target	Forward primer (5ʹ–3ʹ)	Reverse primer (5ʹ–3ʹ)
ITGα1	GGTTCCTACTTTGGCAGTATT	AACCTTGTCTGATTGAGAGCA
ITGα2	GGAACGGGACTTTCGCAT	GGTACTTCGGCTTTCTCATCA
ITGα5	TGCAGTGTGAGGCTGTGTACA	GTGGCCACCTGACGCTCT
ITGα6	TTGAATATACTGCTAACCCCG	TCGAAACTGAACTCTTGAGGATAG
ITGβ1	GAAGGGTTGCCCTCCAGA	GCTTGAGCTTCTCTGCTGTT

### Cell adhesion assay

Cells were seeded in 96-well plate at 37°C with 5% CO_2_ for 24 hr. Cells were washed with PBS and dissociated by trypsin-EDTA (0.05%) 37°C for up to 30 min. Trypsinization was terminated with medium containing 10% FCS. Cell numbers were then quantified using the CyQUANT NF Cell Proliferation Assay Kit (Thermo Fisher Scientific). Cells were incubated with 50 µl of Hanks balanced salt solution containing 0.1% dye and 0.1% delivery reagent at 37ºC for 30 min. The fluorescence intensity was measured using excitation and emission wavelength at 485 nm and 530 nm, respectively.

### Wound healing assay

The migration of cells was investigated by wound healing assay. Parent and NAT1 KO MDA-MB-231 cells (5 x 10^5^) were seeded in a six-well plate in triplicates and incubated for 2 days at 37ºC. Scratches were generated using 1 ml micropipette tip when cells were reaching 100% confluence. Cells were then washed twice with PBS and incubated at 37ºC in complete medium. Images were taken after 0 hr and 20 hr and cell areas were quantified by using ImageJ (MRI Wound Healing Tool). Cell migration was defined as [Cell area (20 hr) – Cell area (0 hr)]/Cell area (0 hr).

### Xcelligence real-time cell analysis (RTCA) migration and invasion assay

Migration and invasion were quantified using the xCELLigence RTCA DP instrument. For cell migration, parental and NAT1 KO cells were seeded in RPMI-1640 medium containing 10% FCS for up to 7 hr. Measurements of impedance (cell index) were performed every 15 min. For the invasion assay, Matrigel (#354230, Corning) was diluted 1:40 with serum-free medium and 50 µl was transferred to the CIM-16 upper chamber to form a thin layer. The coated upper chamber was incubated at 37ºC for 4 hr. Medium (160 µl) containing 20% FCS was added to the lower chamber before the pre-coated upper chamber was assembled. After adding 30 µl serum-free medium to the upper chamber per well, the plate was equilibrated for 60 min equilibrium. A background measurement was performed after the incubation. Cells in serum-free medium were applied to each well and incubated at 37°C with 5% CO_2_ Data were collected every 15 min for up to 20 hr.

### Xcelligence real-time cell analysis (RTCA) adhesion assay

Adhesion was performed in an E-Plate 16 using the xCELLigence RTCA DP instrument. Each well was coated with 20 µg/ml collagen at 37ºC for 1 hr followed by washing with PBS. The wells were blocked with 0.5% (w/v) bovine serum albumin (BSA) in PBS at 37ºC for 20 min. The chambers of the plate were then washed again with PBS and 50 µl serum-free medium was added. The plate was locked in a cradle pocket of the RTCA DP Analyser for an initial background measurement. Cells (1.5 x 10^4^ in 100 µl) in serum-free medium were added and impedance was measured every min up to 60 min.

### Confocal microscopy

Collagen (Roche) at 20 µg/ml was coated onto sterile coverslips at 37ºC for 1 hr in 12 well plates. After washing with PBS, coverslips were blocked with 0.5% BSA in PBS for 20 min. Cells (1.5x10^4)^ were seeded onto each coverslips for 20 hr. The cells were then fixed with 4% paraformaldehyde/cytoskeletal buffer (100 mM KCl, 300 mM sucrose, 2 mM EGTA, 2 mM MgCl2, 10 mM PIPES, pH 7.4) for 20 min, then washed three times with PBS. Coverslips were incubated with 0.05% Triton X/PBS solution for 5 min and washed with PBS. BSA (3%) in PBS was used to block the coverslips for 1 hr. Phalloidin was diluted with 3% BSA and incubated with the cells at 4ºC overnight. After three washings with PBS, coverslips were mounted with ProLong Diamond Antifade Mountant with DAPI (P36962, Thermo Fisher Scientific) and images were acquired with Olympus FV1000 upright confocal microscope. To quantify cell morphology (cell area and aspect ratio), individual cells were analyzed using ImageJ software. A minimum of 60 images were analyzed for each cell-line

### Western blot

For Western blot analysis of cell culture medium, cells were seeded in six-well plates at 5 × 10^5^ cells/well and cultured for 24 hr in medium containing 10% FCS. The medium containing 10% FCS was removed and replaced by serum-free medium overnight. The medium was recovered and filtered using an Amicon Ultra-2 Centrifugal Filter (Merck Millipore) and boiled in Laemmli sample buffer for 5 min. Samples were then electrophoresed at 200 V on 12% SDS-polyacrylamide gels for 45 min, transferred to nitrocellulose membranes for 1 hr at 350 mA, blocked with 5% skim milk in PBS containing 0.05% Tween-20 (PBST) for 1 hr and incubated with anti-MMP1 (ab52631, Abcam), anti-MMP3 (ab53015, Abcam) or anti-MMP9 (ab76003, Abcam) overnight at 4ºC. Membranes were stained with horseradish peroxidase-conjugated secondary antibodies for 1 hr at room temperature and then developed using Westar ETA C 2.0 (Cyanagen). Imaging was performed using a Kodak image station 4000s pro. Protein expression was quantified by densitometry using ImageJ software.

### Flow cytometry

Cells (80% confluence) were dissociated using Versene Solution (#15,040,066, Thermo Fisher Scientific), washed with PBS and incubated with mouse antibody against human integrin αv (#MABT207, Merck) for 30 min at 4ºC. Cells were then washed three times with PBS and incubated with Alexa Fluor 488 Goat anti-mouse IgG (A-11,001; Thermo Fisher Scientific) for 20 min at 4ºC. Flow cytometry was performed using a FACSCanto flow cytometer (BD Bioscience).

### Statistical analysis

Statistical analysis was performed using GraphPad Prism 8 software. Data are presented as mean ± s.e.m. Differences in mean values between multiple groups were analysed using one/two-way ANOVA and Student’s *t*-test. P-value < 0.05 was determined as significant.

## Supplementary Material

Supplemental Material
