# Supplementary figures and images for: Effect arylamine N-acetyltransferase 1 on morphology, adhesion, migration, and invasion of MDA-MB-231 cells: role of matrix metalloproteinases and integrin αV
Source: Cell Adh Migr. 2020 Jan 7;14(1):1–11. doi: 10.1080/19336918.2019.1710015 (PMC6961680; doi:10.1080/19336918.2019.1710015)

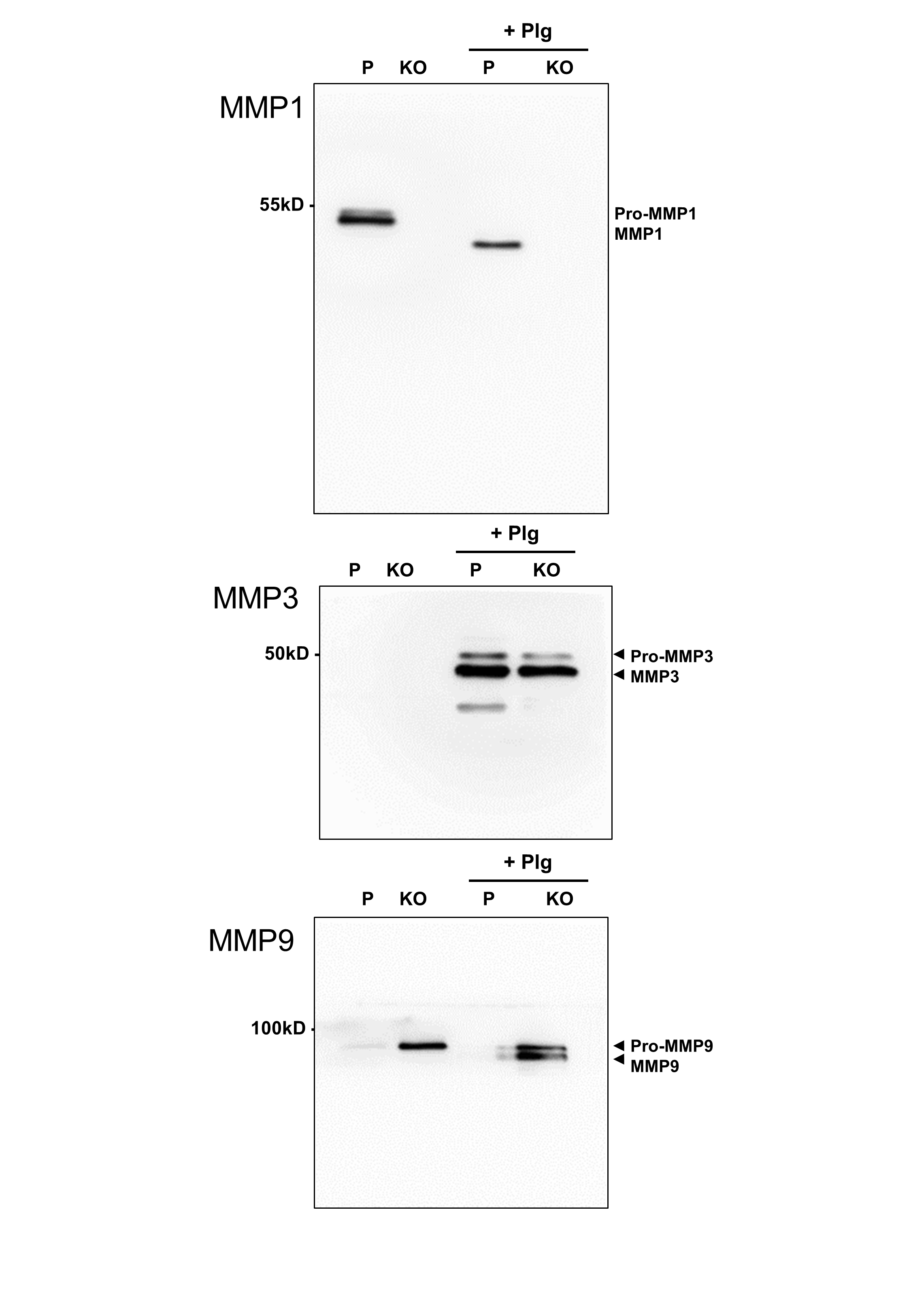

Supplement: Supplemental Material [file kcam-14-01-1710015-s001.tif]
